# Intersensory attention deficits in schizophrenia relate to ongoing sensorimotor beta oscillations

**DOI:** 10.1038/s41537-025-00571-8

**Published:** 2025-02-17

**Authors:** James Kenneth Moran, Daniel Senkowski

**Affiliations:** Department of Psychiatry and Neurosciences, Charité - Universitätsmedizin Berlin, corporate member of Freie Universität Berlin and Humboldt Universität Zu Berlin, Charité Campus Mitte (CCM), Charitéplatz 1, 10117 Berlin, Germany

**Keywords:** Schizophrenia, Biomarkers

## Abstract

This study tested whether intersensory attention deficits in people with schizophrenia (SZ) relate to aberrant ongoing oscillations in sensory cortices. Electroencephalography (EEG) was recorded while individuals with schizophrenia (*N* = 27) and healthy controls (HC; *N* = 27) performed a visual-tactile target detection task. Ongoing alpha (8–12 Hz) and lower beta (13–20 Hz) band oscillations in visual and sensorimotor cortices were examined. Behavioral data suggested an intersensory attention deficit in patients. EEG data revealed stronger alpha-band oscillations for tactile vs. visual attention conditions in the visual cortex of both study groups. In the sensorimotor cortex contralateral to the tactile stimulation site, patients showed an additional intersensory attention effect in ongoing beta-band oscillations, which was negatively related to cognitive and positive symptoms of the PANSS. Our findings extend previous results from unisensory attention research and suggest that deficits in intersensory attention and alterations in sensorimotor beta oscillations are related to schizophrenia symptomatology.

Attention deficits are common in patients with schizophrenia (SZ) and they have a negative impact on daily functioning^1^. To date, studies of attention in SZ have primarily used unisensory stimulation protocols, including auditory-only^2^ or visual-only^3^ stimuli. However, real-world situations typically involve input from multiple sensory modalities and this input must be selected and prioritized, requiring intersensory attention (IA)^4^. IA refers to the ability to selectively attend to stimuli from one sensory modality while ignoring stimuli from other sensory modalities. Thus far, only a few studies have investigated IA processing in SZ^5–8^. These studies have focused on the effects of IA on stimulus processing, but they have not investigated whether there are deficits in ongoing attentional processing. As alterations in ongoing attention have been found in unisensory experimental setups^9–11^, it is possible that patients with SZ also have deficits in ongoing processing of IA.

Electrophysiological studies of ongoing IA in non-psychiatric individuals have shown an important role of neural oscillations, especially in the alpha (8–12 Hz) and beta (13–30 Hz) bands^12–17^. In an experimental paradigm, in which participants were cued to attend to either visual or tactile input, Pomper et al. ^15^ found cue-related IA effects on neural oscillations that were localized in sensory cortices. In the visual cortex, attending to tactile compared to visual input was associated with enhanced alpha and beta band oscillations. In the sensorimotor cortex, IA effects were found only in the beta band, with stronger cue-related beta suppression in attend-tactile compared to attend-visual trials. Attention effects on alpha and beta band oscillations have also been observed in the ventral and dorsal attention networks in a visual-auditory cueing paradigm^16^. Thus, in studies requiring IA, alpha and beta activity can serve as neural marker of attentional processing.

Research in individuals with SZ has shown aberrant neural oscillations in various experimental paradigms and across different frequency bands^18^. This includes changes in alpha band oscillations during ongoing visual attention^19^ and abnormal attentional load effects on beta band oscillations in a visual oddball paradigm^20^. There is also evidence for changes in alpha^21^ and beta band^22^ oscillations during multisensory integration. Hence, neural oscillations seem to play a role in IA. Furthermore, there are alterations in alpha and beta band oscillations that are associated with attentional and integrative multisensory processing in SZ. Therefore, alterations in ongoing oscillations, especially in the alpha and beta band, could also be related to IA deficits in SZ.

To address this question, we recorded neural oscillations using high-density electroencephalography (EEG) from patients with schizophrenia and healthy control participants in a visual-tactile attention paradigm. Using a virtual electrode analysis approach from our previous paper^15^, we examined ongoing, i.e., prestimulus oscillations in the visual and sensorimotor cortex, while attention was directed either to visual or tactile input. Consistent with reports of cue-related IA effects on alpha and beta band oscillations^15,16^, we focused the analysis on these two frequency bands. In addition, we explored whether any IA-related deficits in neural oscillations relate to the task performance, cognitive performance and symptomatology in patients.

## Methods

The effects of IA on poststimulus event-related potentials (ERPs) from this dataset have been reported elsewhere^6^. The focus of the current analysis was on attention effects on ongoing neural oscillations in the prestimulus interval, which differs from our previous analysis. Here we recapitulate the methodological details regarding the study groups, the analysis of behavioral data, and EEG recordings.

### Sample and clinical data

A total of 29 individuals with SZ were recruited at the outpatient units of the Charité Universitätsmedizin, Berlin. They were diagnosed according to the diagnostic and statistical manual of mental disorders, fifth edition (DSM-5)^23^, by experienced psychiatrists at the recruiting institution. Potential recruits taking following substances were excluded, due to the distorting effect upon the EEG signal^24^: benzodiazepines; valproic acid; lithium; haloperidol; and SSRIs. Study groups were matched for handedness^25^, education, smoking^26^, age, and gender. Potential HC recruits were screened for comorbid psychopathology using the German version of the Structured Clinical Interview for DSM-4-RT Non-Patient Edition^27^. Those with immediate family members with a psychiatric or neurological disorder were excluded. Of the total of 58 participants, two from each group were excluded from the behavioral data, as their results indicated that they had not performed the task correctly.

Table 1 provides an overview of demographic and symptom profiles of HC and SZ groups, showing that matching criteria were maintained in the final sample (SZ = 27 [10 female, mean age = 38.78], HC = 27 [11 female, mean age = 39.63]). Symptom severity in SZ was assessed with the PANSS^28^, with items grouped according to the five-factor model^29^. The interview was always carried out by only one of the team (JKM) to avoid problems of interrater reliability. The cognitive capacity of all participants was tested with the Brief Assessment of Cognition in Schizophrenia (BACS)^30^. All participants were screened for drug use on the day of measurement (Drug-Screen Multi 5 Test, Nal von Minden, amphetamine, benzodiazepine, cocaine, opioids, and cannabis). Written informed consent was obtained from every individual after a comprehensive description of the research. The study was carried out in line with the 2008 Declaration of Helsinki and was approved by the Ethics Commission of the Charité-Universitätsmedizin, Berlin (Approval number: EA1/169/11).

**Table 1:** Demographics for participants.

Age	SCZ m (*N* = 27)	SCZ sd	HC m (*N* = 27)	HC sd	*t*	*p*
°	39.78	9.07	38.63	9.64	−0.45	0.65
Cigarettes (Fagerström)	SCZ med (*N* = 15)	SCZ iqr	HC med (*N* = 13)	HC iqr	w	p
	6	1.5	2	4	32	0.002**
Gender	SCZ	HC				
*female*	10	11				
*male*	17	16				
Education (years)	SCZ med (*N* = 23)	SCZ iqr	HC med (*N* = 23)	HC iqr	w	*p*
	10	3	13	3	299.5	0.424
Antipsychotic Dose	SCZ m (*N* = 27)	SCZ sd			Count:	
	83%†	57%			Clozapine	8
					Amisulpride	7
					Quetiapine	3
					Olanzapine	6
					Aripiprazole	7
					Risperidone	6
					Ziprasidone	3
					Paliperidone	1
BACS Z scores	SCZ med (*N* = 27)	SCZ iqr	HC med (*N* = 27)	HC iqr	w	*p*
BACS Z Verbal	−0.94	1.55	0.30	2.02	541	0.002**
BACS Z Digit	−0.26	1.14	−0.05	1.13	460	0.100
BACS Z Token	0.12	1.57	0.43	1.68	444.5	0.169
BACS Z Fluency	−0.62	1.09	0.08	1.26	516	0.009**
BACS Z Symbol	−1.18	1.53	0.26	1.33	555.5	0.001***
BACS Z Tower	0.34	0.61	0.19	0.50	394	0.876
BACS Composite Z-Score	−0.79	1.69	0.21	0.95	553	0.001***
PANSS	SCZ m (*N* = 27)	SCZ sd				
Positive 5	7.67	4.12				
Negative 5	12.30	5.78				
Disorganized 5	6.19	2.48				
Excitement 5	12.15	3.83				
Depression 5	7.15	3.57				

Differences between groups were calculated by either parametric independent *t*-tests (*t*) or non-parametric Wilcoxon tests (w), where data did not fulfill assumptions of parametric tests, *m* mean, *med* median, *sd* standard deviation, *iqr* inter-quartile ratio. Antipsychotic dose is calculated as the percentage of standard daily dose of Olanzapine, which is 10 mg. BACS raw scores were converted to z-scores normalized for age and gender. SD refers to standard deviation.

*p < 0.05, **p < 0.01, ***p < 0.001.

^†^percentage of standard daily dose of Olanzapine.

### Setup and procedure

**Fig. 1:**
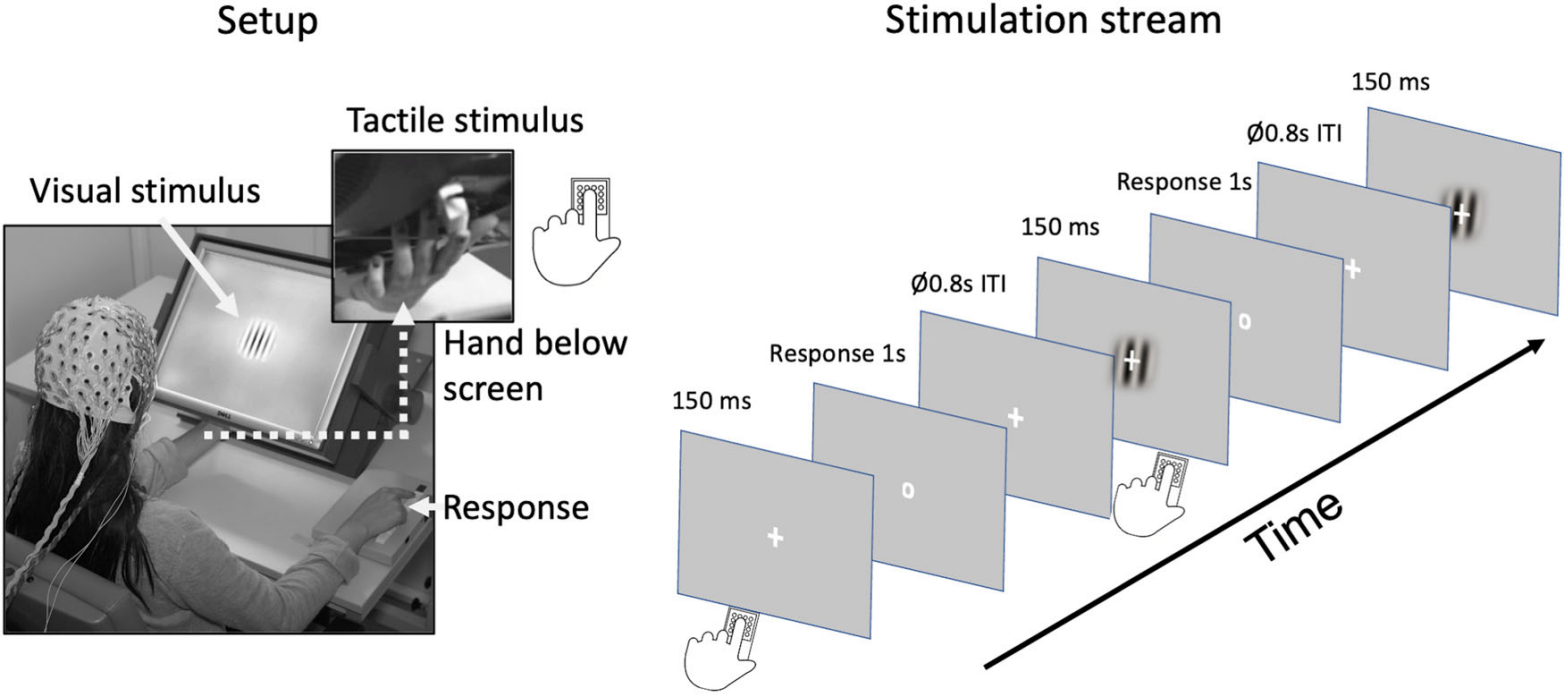
Setup of the intersensory attention paradigm and stimulation stream. Left panel: Setup of the study. The tilted monitor enabled simultaneous and spatially aligned presentation of visual stimuli (central location on the screen) and tactile stimuli (presented via a Braille-stimulator placed on the back of the screen and applied to the index finger of the left hand). Muscle fatigue was prevented by cushioning the left hand. Behavioral responses were given by the index finger of the right hand. Right panel: Stimulation stream comprising of unisensory visual, unisensory tactile and bisensory visuotactile standard and target stimuli, which were presented in random order. Participants attended to either visual or tactile input, pressing the response button when the occasional target stimulus in the attended sensory modality appeared.

Participants performed the experiment in an electrically and acoustically shielded chamber with low lighting. Three stimulus categories were presented in random order: unisensory-visual (V), unisensory-tactile (T), and bisensory (VT). An occasional target was presented in either visual or tactile modality. Visual stimuli were presented centrally against a gray background, (luminance 30 cd/m^2^) on a tilted thin-film transistor display (TFT) monitor (Fig. 1). The visual standard stimulus was a Gabor patch, within a circular form (diameter: 5.75°, spatial frequency = 1 cycle per degree, Gaussian standard deviation [SD] = 2°, duration = 150 ms). The target stimulus differed from this in flickering at 16.7 Hz for the full 150 ms duration of presentation. Tactile stimuli were presented at the same location as the visual stimuli, which ensured that spatial attention was comparable in the visual and the tactile attention conditions. Tactile stimuli were delivered with a piezoelectric Braille stimulator (QuaeroSys) that was placed on the back of the monitor. The stimulator had 16 pins arranged in a square, with 2.5 mm distance between each. The standard tactile stimulus was a short pulse of 150 ms delivered to the left index finger of the participant. The target tactile stimulus consisted of rhythmic elevations and contractions of the pins at 16.7 Hz for 150 ms. Visual and tactile stimuli were presented simultaneously during bisensory VT trials. Bisensory target stimuli were always either both standard or both target, there was no target/standard combination. To mask the sound of the Braille stimulator, white noise was presented over headphones, adjusted prior to the beginning of the experiment, to ensure participants could not hear the Braille stimulator.

Participants familiarized themselves with the task in several trials prior to the experimental runs. Responses were registered with a button press of the right index finger when a target was present. The response period was cued with the fixation cross transforming into a circle (response cue) at the end of the stimulation, i.e., after 150 ms (Fig. 1, right panel). The intertrial interval varied between 1600 – 2000 ms (average 1800 ms).

Top-down attention to the visual and tactile stimuli was altered blockwise, so that the attention of the participant was focused either on visual or tactile input for the length of the block. In half of the blocks (attend-visual condition), participants had to detect targets in the visual modality (and ignore tactile stimuli) and in the other half (attend-tactile condition), they had to detect targets in the tactile modality (and ignore visual stimuli). Participants received information about the attended modality before each block. In total, there were 1722 trials across 14 blocks (123 trials per block), with each block lasting about 4 min. This breaks down into 235 standard trials for V, T and VT stimuli, and an additional 52 V, T, and VT target stimuli. This is the case for both attention conditions. Hence, target trials made up about 18% of trials overall. The trial order was randomized for each individual participant.

### EEG recording and data preprocessing

EEG data was recorded with a 128-channel passive EEG system (EasyCap), including two electrooculography (EOG) electrodes (online: 1000 Hz sampling rate, with a 0.016–250 Hz bandpass filter; offline: 49–51 Hz 4th order Butterworth notch filter, 125 Hz 24th order finite impulse response (FIR) lowpass filter, downsampled to 500 Hz, 1 Hz 1500th order FIR highpass filter). The online nasal reference was transformed to average reference offline. Trials containing nonstationary artifacts were manually removed. After this, there was no evidence of difference between HC and SZ groups in number of trials remaining (HC: M = 1055.93; SD = 179.67, SZ: M = 1057.63, SD = 138.51; *t*(48.84) = −0.04, *p* = 0.969; BF10 = 0.27). EOG and ECG artifacts were subsequently identified with Independent Component Analyses^31^^,32^. The median number of components cut for HC and SZ groups was 3. Spherical interpolation was employed to fill in remaining noisy channels. The number of channels remaining from the original 126 was also comparable between groups (SCZ = 118.14 [SD = 5.25] channels; HC = 116.48 [SD = 4.50] channels, *t*(51.18) = − 1.27, *p* = 0.209; BF10 = 0.53).

### Statistical analyses

Prior to the analyses of differences in attention effects between the two study groups, we examined whether the effects of IA on ongoing alpha and beta oscillations in the HC group alone were similar to those previously found in cue-related IA paradigms^15,33^. Specifically, we predicted IA effects on alpha oscillations in the visual cortex and on beta oscillations in the sensorimotor cortex.

Next, linear mixed-effects models (LMEs) were computed to examine the effects of the factors Group (HC, SZ), Attention (attend-visual, attend-tactile), and sensory Modality (unisensory, bisensory) as well as their interactions on outcome variables for both oscillatory (relative alpha and beta band power) and behavioral outcomes (d-prime and reaction times). Fixed effects included the main effects of Group, Attention and Volume of Interest (VOI) as well as their two-way and three-way interactions. Random intercepts were included for participants to account for individual variability. Furthermore, sum-to-zero contrasts were used for categorical predictors to allow comparisons relative to the grand mean. The linear mixed-effects model was fitted using restricted maximum likelihood estimation (REML) with the lme4 package (version 1.1.35.5) in R. Fixed effects were assessed using a Type III Wald F-statistics over the anova() function, which evaluates the significance of individual predictors in the presence of random effects. Standardized beta (ß) coefficients were calculated using the parameters package (version 0.23.0). Significant main effects and interactions were further explored with the emmeans R package (version 1.10.4) and adjusted means (M-adj) or mean differences (M-diff) are presented with confidence intervals, t-statistics, Bonferroni corrected *p*-values, and Bayes Factors.

### Bayes factor

For the interpretation of the statistical results, we calculated Bayes Factors (BF10)^34^; to estimate the strength of evidence for both H1 and H0, using the Pingouin package in Python3^35^. A BF10 of 1 indicates equivocal evidence for H1 or H0. BF10 from 1 – 3, from 3 – 10 and above 10 indicate, respectively anecdotal, moderate and strong evidence for H1. Conversely, BF10s from 1/3 to 1, 1/10 to 1/3, and below 1/10 indicate respectively anecdotal, moderate and strong support for H0^36^. Since we had no prior assumptions about the size of the effects tested, we used the default setting in Pingouin of the Cauchy Scale at 0.707^37^.

### Analysis of behavioral data

The behavioral findings for this dataset have been previously presented elsewhere^6^. The results are relevant to our new focus on prestimulus oscillations, so are presented again here for clarity. Participants’ responses, both correct and false were combined into d-prime values^38^. Correct responses were defined as a response to a target in the attended modality, and false alarms were defined as a response to a standard stimulus in the attended modality. Mean percentage of responses outside of the predefined analysis interval of 100–900 ms following the response cue were 3.8% for HC and 7.45% for SZ (t(40) = −2.14, *p* = 0.039, BF10 = 1.81). Additionally, reaction times (RT) were analysed across different conditions and groups.

### Source analysis

**Fig. 2:**
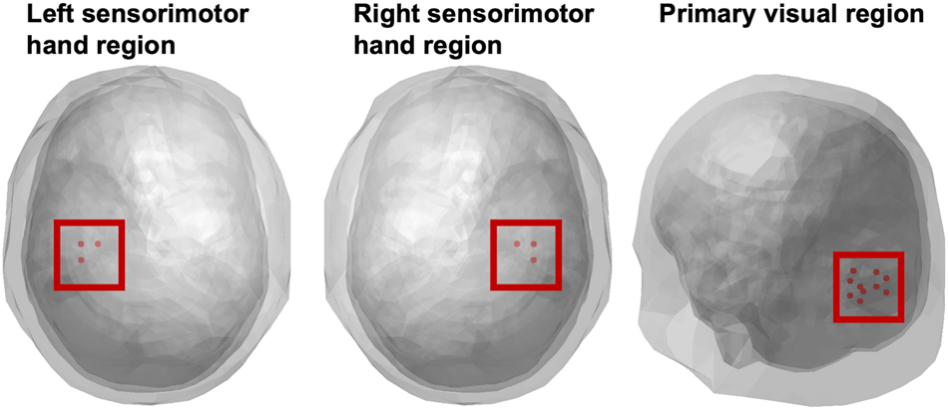
Virtual electrodes with the corresponding nodes (dots) for each of the three VOIs. From left to right: left sensorimotor hand region, right sensorimotor hand region (each with 3 nodes), viewed from axial plane, and primary visual region (11 nodes), left posterior view.

As with our previous visual-tactile attention study in healthy individuals^15^, the source space analysis of neural oscillations focused on three VOIs from the BrainMap atlas^39^: (1) The primary visual cortex, (2) the right sensorimotor hand area, i.e., contralateral to the tactile stimulation, and (3) the left sensorimotor hand area. A set of virtual electrodes matching these volumes was derived from a three-dimensional grid with 1 cm resolution, encompassing the brain volume^40^. The left and right sensorimotor hand areas contained 3 nodes, and the visual VOI contained 11 nodes (Fig. 2). Raw data was projected onto virtual electrodes (nodes) with corresponding spatial filters for each participant and grid point employing a realistic three-shell boundary-element volume element based on the MNI standard brain (MNI; http://www.mni.mcgill.ca). A common filter was created across both conditions using the covariance matrix of the averaged single trials at sensor level and the corresponding leadfield. The filter was calculated for the following interval of ±1000 ms around the stimuli. To provide the beamformer with more data (to obtain a more accurate source estimation) this interval is longer than the interval used for the statistical analysis. The lambda regularization parameter was 5%. Single trials were projected through these filter parameters for each subject, condition and virtual electrode.

### Analysis of neural oscillations

For the time-frequency analyses, we used a Hanning taper for a frequency range of 2 – 40 Hz (0.5 Hz intervals) with a window length of 4 cycles at each frequency. Our prestimulus time of interest was between -800 ms and -200 ms for all possible trials (visual, tactile, visuotactile; both standards and targets), which is identical to the analysis window used in our previous attentional visual-tactile cuing study in healthy individuals^15^. Consistent with the findings of that previous study^15^, we expect IA effects in the lower beta band and therefore choose the often used 13–20 Hz range for the analysis of lower beta oscillations. Similarly, alpha was defined as 8–12 Hz, to be comparable with previous findings, and provide a common standard of comparison across groups. To study attention effects in the current study, we calculated the relative difference in oscillatory power between attend-visual and attend-tactile conditions. To this end, we subtracted the power in the tactile condition from the visual condition, and then divided the difference by the tactile power, multiplying by 100 to attain an index of relative percentage change across attention conditions (((V – T)/T)*100). The effect of Group (HC vs. SZ) and VOI were analyzed in a linear mixed effects model, with individual as random effect. As in previous M/EEG studies investigating IA^15,41,42^, the analysis was performed separately for alpha and beta activity.

### Relationships between neural oscillations with behavior, cognitive performance and clinical parameters

In regions where there were significant differences between SZ and HC, we tested the relationships between neural oscillations and measured behavioral parameters, namely d-prime, RT, BACS performance and the 5 dimensions of the PANSS scores (positive, negative, cognition, depression, excitation) in the SZ group. Potential confounds (medication, i.e. antipsychotic dose, and cigarette consumption) did not show evidence of association with neural oscillations (all *p* > 0.05, BF10 < 1).

## Results

### Behavioral data

#### d-prime

**Fig. 3:**
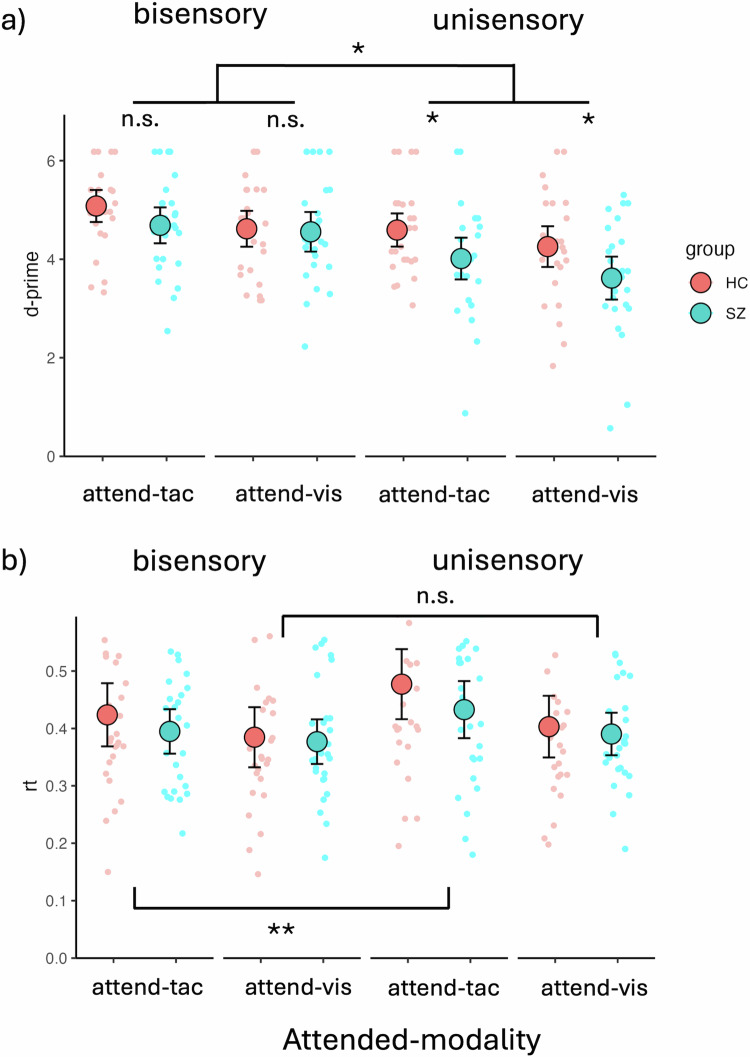
Behavioral results for d-prime values and reaction times. **a** Behavioral data showing deficits in IA for unisensory visual and unisensory tactile stimuli but not bisensory VT stimuli. The figure contrasts individuals with SZ (SZ, turquoise dots) and HC (red dots). Unisensory deficits were present in both visual and tactile attention conditions. There was an overall better performance for bisensory stimuli than for unisensory stimuli across groups and sensory modalities. People with SZ performed worse than HC specifically on the unisensory stimuli, whereas there were no significant performance differences on bisensory stimuli. **b** Behavioral data showing RT differences for bisensory vs. unisensory stimuli. In the attend-tactile blocks, differences between bisensory and unisensory stimuli were found, with bisensory stimuli showing faster RTs than unisensory tactile stimuli. This was not apparent for the attend-visual blocks. The large dots represent mean values with the error bars representing ± 1SE. The smaller dots represent individual datapoints. Significant main effects and interactions are represented with asterisks (n.s, *p* > 0.05, *p < 0.05, **p < 0.01).

The LME model analysis of d-prime values revealed a main effect of sensory Modality (F(1,156) = 46.59, *p* < 0.0001, *β* = 0.29), with higher d-prime values for bisensory visuotactile stimuli (adj-M = 4.74 [4.50, 5.00]) compared to unisensory stimuli (adj-M = 4.12, [3.88, 4.36]; t(156) = 6.83, *p* < 0.0001, BF10 > 100). There was no main effect of Group (F(1,52) = 3.53, *p* = 0.066, β = 0.20), with HC (adj-M = 4.64, [4.32, 4.95]) not being statistically different from SZ (adj-M = 4.22, [3.90, 4.54]; *t*(52) = 1.88, *p* = 0.066, BF10 = 1.16). However, there was a main effect of Attention (F(1,156) = 13.52, *p* < 0.0003, *β* = 0.15), with tactile stimuli (adj-M = 4.59, [4.35, 4.83]) showing higher d-prime values than visual stimuli (adj-M = 4.26, [4.02, 4.50]; *t*(156) = 3.68, *p* = 0.0003, BF10 = 50.99). There was also a significant interaction between Group and sensory Modality (F(1,156) = 4.48, p = 0.036, *β* = −0.09), with a significant difference between HC (adj-M = 4.43, [4.09, 4.76]) and SZ (adj-M = 3.82, [3.48, 4.15]; *t*(69.8) = 2.54, p = 0.0135, BF10 = 3.66) for unisensory stimuli, but no evidence of a difference between HC (adj-M = 4.85, [4.51, 5.19]) and SZ (adj-M = 4.62, [4.28, 4.96]; t(69.8) = 0.95, *p* = 0.348, BF10 = 0.40) for bisensory stimuli. The difference of differences for these two contrasts was also significant (unisensory minus bisensory = −0.38, t(156) = −2.12, p = 0.0358, BF10 = 1.70). This overall indicates that people with SZ have impaired unisensory processing relative to HC and suggests IA deficits in patients (Fig. 3a). The criterion of responses did not show evidence of differences between groups on any of the four response categories measured with non-parametric Mann-Whitney-U tests. Bisensory visual [Med_sz_ = 2.71 [IQR = 0.76], Med_hc_ = 2.71 [IQR = 0.95]; W = 354, *p* = 0.86, BF10 = 0.30]; bisensory tactile [Med_sz_ = 2.71 [IQR = 0.61], Med_hc_ = 2.71 [IQR = 0.81]; W = 434.5, *p* = 0.74, BF10 = 0.74]; unisensory visual [Med_sz_ = 2.48 [IQR = 1.26], Med_hc_ = 2.71 [IQR = 1.25]; W = 374.5, *p* = 0.87, BF10 = 0.27]; unisensory tactile [Med_sz_ = 2.72 [IQR = 0.81], Med_hc_ = 2.72 [IQR = 0.69]; W = 365, p = 1, BF10 = 0.30].

#### Reaction Time (RT)

The LME model analysis of RTs revealed a main effect of Attention (F(1,156) = 393.42, *p* < 0.0001, *β* = 0.17), with longer RTs for tactile targets (*m* = 0.43, [0.40, 0.47]) compared to visual targets (m = 0.39, [0.36, 0.42], *t*(156) = 6.72, *p* < 0.0001). There was no main effect of Group (F(1,52) = 0.52, *p* = 0.475, β = 0.09), but a main effect of sensory Modality (F(1,156) = 198.43, *p* < 0.0001, *β* = −0.12). Follow-up contrasts showed faster responses for bisensory (adj-M = 0.40, [0.36-0.43]) over unisensory stimuli (adj-M = 0.43, [0.39, 0.46], *t*(156) = −4.46, *p* < 0.0001, BF10 > 100). In addition, there was a significant interaction between Attention and Modality (F(1,156) = 46.44, *p* = 0.033, *β* = −0.06). Follow-up Bonferroni adjusted contrasts showed that in the attend-tactile condition, RTs to bisensory stimuli were significantly shorter (adj-M = .41, [0.38, 0.44]) than RTs to unisensory tactile targets (adj-M = 0.46, [0.42, 0.49], t(156) = −4.67, *p* < 0.0001, BF10 > 100). This was not the case for the attend-visual condition in which there was no difference in RTs between unisensory (adj-M = 0.40, [0.36, 0.43]) and bisensory (adj-M = 0.38, [0.35, 0.42]; *t*(156) = −1.63, *p* = 0.106, BF10 = 0.81) stimuli. The difference of differences for these two contrasts was also significant (attend-visual minus attend-tactile = −0.03; *t*(156) = −2.15, *p* = 0.033, BF10 = 1.79). This overall suggests an elevated difficulty in the attend-tactile compared to the attend-visual condition, which especially affected RTs to unisensory stimuli (Fig. 3b).

### Oscillatory responses

**Fig. 4:**
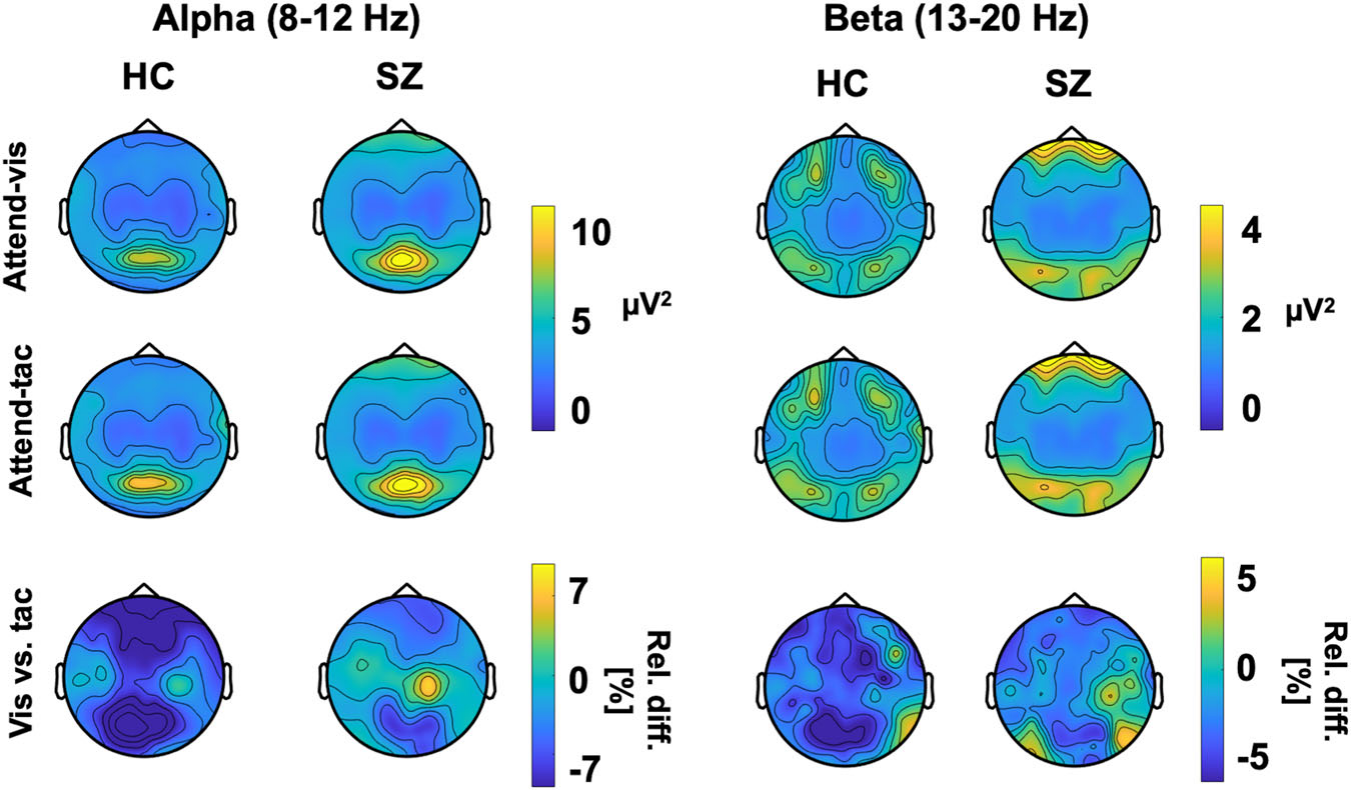
Topographical plots of alpha band and beta band activity at the surface electrode level. Plots show alpha (left) and beta (right) power for HZ and individuals with SZ for each attention condition as well as the relative percent difference between visual and tactile attention conditions.

Figure 4 depicts topographic plots of the surface electrode activity for ongoing alpha (8–12 Hz) and beta (13–20 Hz) activity. This overview of activity shows the strongest ongoing alpha oscillations over occipital and sensorimotor regions. For the beta oscillations, the SZ group shows the strongest variation over sensorimotor regions. These are tested in more detail in the virtual electrodes analyses of occipital and sensorimotor regions that follow. LME models were applied for relative change in alpha as well as beta band activity, testing the effects of Group as well as VOI, with individual as random factor. It is possible that our approach of averaging over the predefined TOI obscured potential dynamic changes in this response. Such changes have been found previously in studies using attentional cues^43,44^, and patients with SZ also have deficits in temporal expectation processing^45^, which may have played a role the current study. To test this, we examined power fluctuations in the prestimulus interval from -800 ms to -200 ms, but did not find systematic patterns of power changes or fluctuations differences in IA effects between groups for either alpha or beta oscillations in the three VOIs (Supplement A).

#### Alpha power

**Fig. 5:**
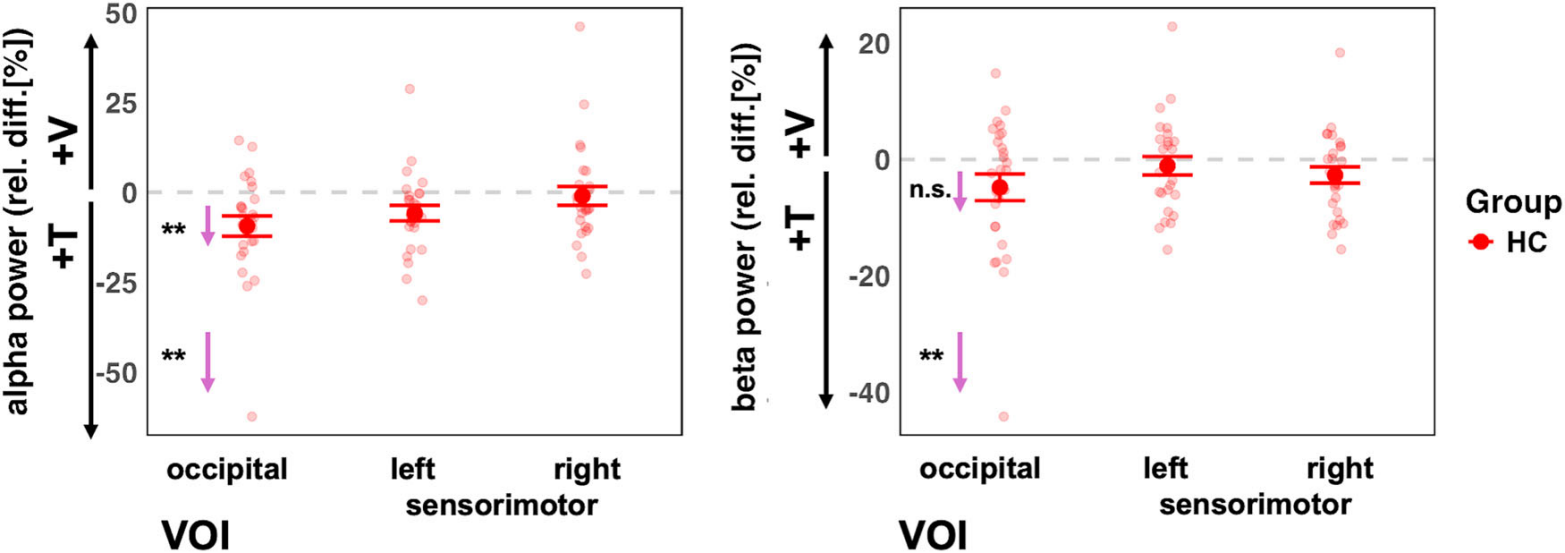
Ongoing alpha (8-12 Hz) and beta (12–20 Hz) power in the control group. Left panel: The control group showed the predicted stronger alpha power in the visual cortex when attention was directed to tactile compared to when it was directed to visual stimuli VOI (left panel). This was the case for when one outlier value was removed. Right panel: There were no significant attention effects on beta power (after removing one outlier value). The small arrows with asterisks represent Bonferroni-corrected follow-up contrasts between the attention conditions, *p < 0.05, **p < 0.01,***p < 0.001 n.s. = non-significant.

The analysis of IA effects on ongoing alpha oscillations in the visual cortex, which we first performed for the control group only, revealed an overall effect of VOI (F(2,52) = 6.93, *p* = 0.002), showing differences between the attention effects across the three different target regions. Follow-up contrasts showed significant IA effects on alpha oscillations for the occipital VOI (adj-M = −9.41 [-14.54, 4.27], *t*(45) = −3.69, *p* = 0.002, BF10 = 54.34) but not for the left sensorimotor VOI (adj-M = −5.87 [-11.00, -0.73], t(45) = −2.30, *p* = 0.078, BF10 = 2.33) or the right sensorimotor VOI (adj-M = −1.01 [-6.14, 4.13], t(45) = −0.39, *p* = 1, BF10 = 0.39). The negative values here indicate that alpha oscillations were stronger in the attend-tactile condition compared to the attend-visual condition. Inspection of the graph (Fig. 5) suggests that this result could have been driven by an extreme value. However, the effects were still present when this value was removed from the analysis [main effect of VOI: (F(2,50) = 5.85, *p* = 0.005) occipital (adj-M = −7.39 [-11.85, 2.89], *t*(47.4) = −3.31, *p* = 0.005, BF10 = 54.31)]. Our finding is in line with previous reports of cue related IA effect on occipital alpha oscillations in healthy individuals^15,33^.

**Fig. 6:**
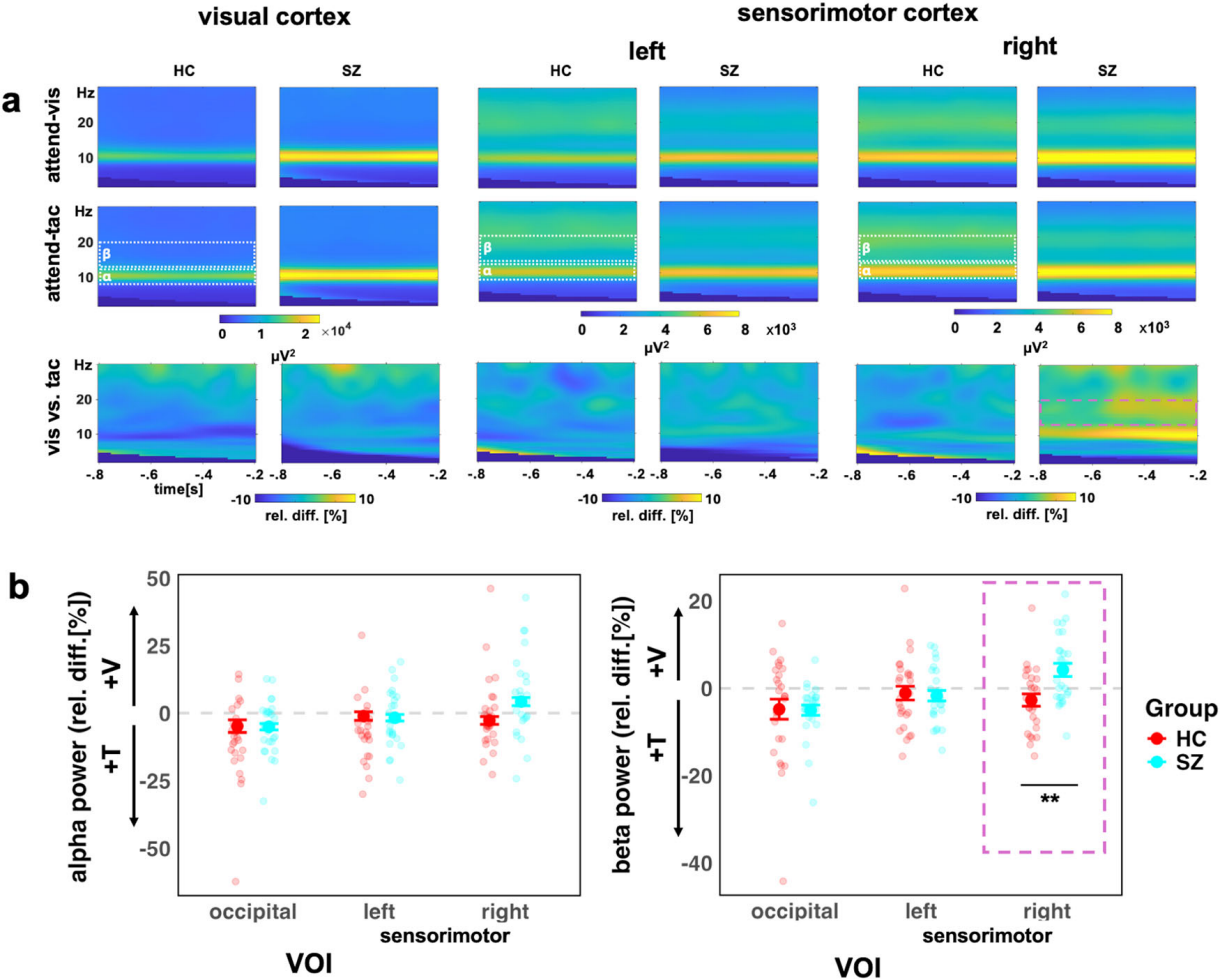
Ongoing alpha (8-12 Hz) and beta (13–20 Hz) oscillations are altered in people with SZ relative to HC participants. **a** Time-frequency representations for the occipital VOI (left), left sensorimotor VOI (middle), the right sensorimotor VOI (right), showing visual-attention (V) and tactile-attention (T) conditions and the contrast between conditions. **b** Alpha (left) and beta (right) power for both study groups (HC, red, and SZ turquoise). The *y*-axis scale represents the relative percentage change between conditions, i.e., (V – T)/T * 100. Values below zero represents a relatively stronger power in attend-tactile condition, whereas a difference above zero represents a stronger power in attend-visual condition. Individual points represent participants, large dots show mean with ± 1 SE CI. The purple box highlights the significant Group*VOI interaction for beta power in the right sensorimotor VOI, **p < 0.01.

Examining both study groups in the LME model analysis which included both groups, we found a main effect of VOI for alpha activity (F(2, 104) = 16.43, *p* < 0.0001), but not for Group (F(1, 52) = 3.23, *p* = 0.078) (Fig. 6). Furthermore, there was no significant interaction between VOI and Group (F(2, 104) = 0.52, *p* = 0.60). Follow-up Bonferroni adjusted contrasts showed that there was a greater relative change in the right sensorimotor VOI, compared to left (diff-M = −6.46, t(104) = −3.69, *p* = 0.001, BF10 = 55.61) and occipital (diff-M = −9.88, *t*(104) = −5.65, *p* < 0.0001, BF10 > 100) VOIs, with no evidence of difference between left and occipital VOIs (diff-M = 3.42, t(104) = 1.96, *p* = 0.16, BF10 = 0.68). It is possible that there are systematic differences in alpha frequency between SZ and HC participants^45^. Therefore, we calculated an additional analysis of the individual alpha frequency. First, we observed that the average alpha frequency was slightly lower in the SZ group (adj-M = 9.91, 9.62-10.20) than HC (adj-M = 10.35, [10.06–10.60]; t(52) = 2.17, *p* = 0.035, BF10 = 1.85). Thus, for the LME model analysis with individual alpha frequency, we observed the same effects as in the analysis of the predefined alpha frequency range (Supplement B).

#### Beta activity

The analysis of IA effects on ongoing beta oscillations in the sensorimotor cortex in the HC group only did not reveal the predicted larger beta activity for attend-visual compared to attend-tactile conditions (m = −2.38, [-5.65, 0.90], t(54.8) = −1.45, p = 0.455, BF10 = 0.52). Furthermore, the left sensorimotor VOI showed no significant effects of attention (adj-M = −0.93, [-4.20, 2.34], t(54.8) = 0.57, p = 1, BF10 = 0.24). Hence, previous findings of cue-related IA effects on sensorimotor beta oscillations[15] could not be found for block-wise IA effects in the present study (Fig. 5). This is detailed as follows. The model’s main effect of VOI was non significant (F(2,52) = 1.77, *p* = 0.180). Examining relative beta activity in occipital regions for HC showed larger beta oscillations in attend-tactile condition compared to the attend-vis condition (adj-M = −4.81, [-8.43, -1.184], t(58.5) = −2.66, p = 0.031, BF10 = 3.69). However, this was outlier driven: when the extreme value was removed, this was no longer significant (adj-M = −3.29, [-6.57, -0.22], t(54.8) = −2.02, *p* = 0.146). In addition, in the control group, there was no significant difference in beta activity between the attend-visual and attend-tactile conditions in the right sensorimotor VOI (*m* = −2.38, [-5.65, 0.90], t(54.8) = −1.45, *p* = 0.455, BF10 = 0.52).

Examining both study groups (Fig. 6) in the LME model analysis, there was a main effect of VOI for beta activity (F(2, 104) = 9.53, *p* = 0.0002), but not for Group (F(1, 52) = 1.55, *p* = 0.219). The Group*VOI interaction was significant (F(2, 104) = 5.23, *p* = 0.007). Follow-up Bonferroni adjusted contrasts showed that the group difference was specific to the right sensorimotor region, with SZ (adj-M = 4.23, [1.11, 7.36]) showing higher relative beta activity than HC (adj-M = −2.71, [-5.83, 0.42], *t*(131) = −3.11, *p* = 0.0023, BF10 = 9.36), whereas left (diff-M = 0.60, *t*(131) = 0.27, *p* = 0.79, BF10 = 0.10) and occipital (diff-M = 0.22, t(131) = 0.10, *p* = 0.92, BF10 = 0.10) regions did not show group differences. The interaction contrasts showed that the sensorimotor group differences were greater than occipital VOIs (diff-M = 7.17, *t*(104) = 2.73, *p* = 0.008, BF10 = 3.62), and that there was no evidence of difference between occipital or left sensorimotor VOIs (diff-M = 0.37, *t*(104) = 0.14, *p* = 0.89, BF10 = 0.11).

##### Right sensorimotor beta activity, d-prime and RT

We tested whether the IA effect in the beta band of the right sensorimotor region could be associated with differences in d-prime and RTs reported above. The relation between the relative change in beta power for visual vs. tactile attention conditions was also analyzed in a linear mixed effects model. The RT was collapsed to a single relative variable, the relative change in RT for (v - t)/t. This was then regressed against beta band relative change ((v – t)/t), Mode (multisensory vs unisensory) and Group. Similarly, d-prime was collapsed into a single variable (v – t/t), for both unisensory and bisensory conditions. This was then regressed against Group, Mode, and IA Effect in the beta band. For both d-prime and RT, the IA effect did not predict d-prime scores, nor did it interact with the other factors (Table 2).

**Table 2:** Linear Mixed Effects model of predictors of Relative reaction time (above) and d-prime (below), including relative beta band activity, task modality (multimodal vs unimodal), and group (HC vs. SZ).

RT predictor	β	F(1,50)	*p*
Beta Change	0.03	0.04	0.84
Mode	0.11	2.41	0.13
Group	−0.15	1.11	0.30
Mode*Beta Change	−0.01	0.03	0.87
Group*Beta Change	−0.17	1.59	0.21
Mode*Group	0.04	0.16	0.69
Beta Change*Mode*Group	0.11	2.10	0.15

d-prime predictor	β	F(1,50)	*p*
Beta Change	−0.17	2.03	0.16
Mode	>0.00	>0.00	0.95
Group	−0.16	1.72	0.20
Mode*Beta Change	0.03	0.08	0.78
Group*Beta Change	−0.01	0.01	0.92
Mode*Group	>0.00	>0.00	0.97
Beta Change*Mode*Group	0.03	0.07	0.79

##### Right sensorimotor beta activity and BACS

Regressing BACS overall scores for both groups against relative beta power in the right sensorimotor cortex showed an overall significant model (*r*^*2*^ = 23.3%, F(3,50) = 6.37, *p* = 0.0010) as well as main effects of the IA effect as a predictor of BACS scores (*β* = −0.06, *t* = −2.28, *p* = 0.027, BF10 = 2.24) and of Group (*β* = −0.95, *t* = −2.99, *p* = 0.0043, BF10 = 9.46), with an interaction between the two variables (*β* = 0.27, *t* = 2.07, *p* = 0.044, BF10 = 1.56). However, this effect was driven by an outlier value (Supplement C). When the outlier was removed from the analysis, the interaction was no longer found (*β* = −0.26, *t* = 1.88, *p* = 0.066, BF10 = 1.56). Therefore, we did not calculate follow-up tests for this interaction.

##### Right sensorimotor beta activity and PANSS dimensions

**Fig. 7:**
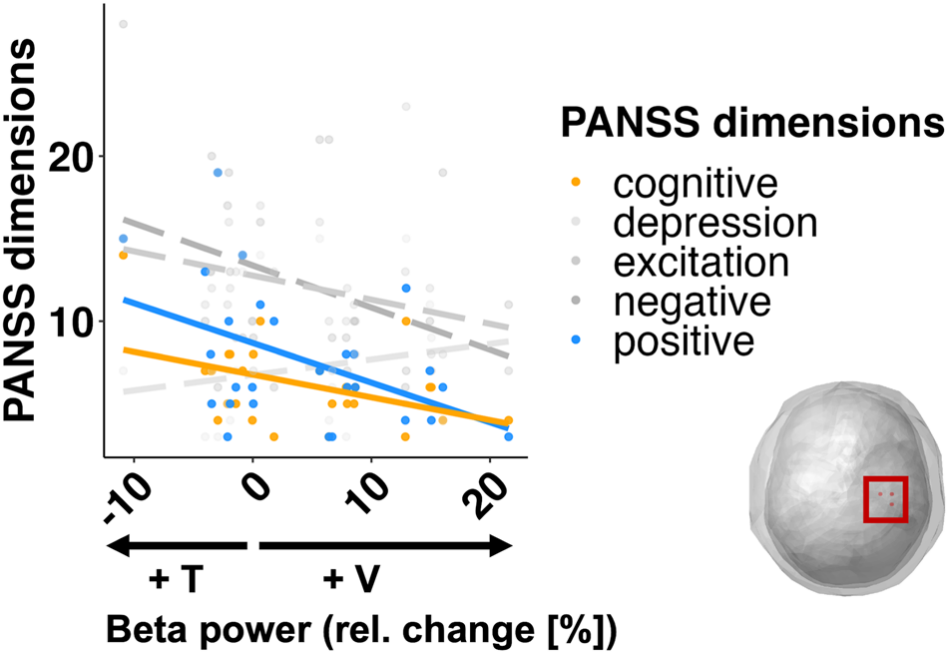
Multivariate multiple linear regression of PANSS dimensions with IA effects on right sensorimotor beta power in patients with SZ. The analysis revealed that a stronger IA effect (i.e. higher beta activity in the non-attended condition) was associated with lower SZ symptomatology in the positive and cognitive dimensions of the PANSS. Other dimensions (in gray) were not significantly predicted by relative beta power change.

For the five dimensions of the PANSS, we carried out a multivariate multiple linear model to ascertain the overall relationship between symptomatology in patients and the attentionally modulated changes in right sensorimotor beta power. The overall model was significant (F(5,21) = 4.31, *p* = 0.0074). Follow-up univariate tests showed that positive symptoms (adj-r^2^ = 17.7%, *β* = −0.24, F(1,25) = 6.6, *p* = 0.0166) and cognitive symptoms (adj-r^2^ = 15.7%, *β* = −0.14, F(1,25) = 5.85, *p* = 0.023) were negatively related to the IA effects (Fig. 7, yellow and blue lines). Thus, a stronger IA effect, i.e., larger differences in beta oscillations between attention conditions, predict lower pathological symptomatology in positive and cognitive dimensions of patients. The relationships between the attention effects on beta activity and the other three dimensions were not significant: (negative (adj-r^2^ = 8.4%, *β* = −0.26, F(1,25) = 3.39, *p* = 0.077); excitation (adj-r^2^ = 5.32%, *β* = −0.15, F(1,25) = 2.46, *p* = 0.129); depression (adj-*r*^*2*^ = 0.4%, *β* = 0.09, F(1,25) = 1.12, *p* = 0.303)).

## Discussion

In this study, we examined the neural mechanisms underlying visual-tactile intersensory attention in healthy controls and patients with SZ. Previously, we reported behavioral deficits and alterations in ERPs for this dataset^6^. Here, we focused on attention effects on ongoing alpha and beta band oscillations in the visual and sensorimotor cortex and their relationship to behavior, cognitive performance and clinical parameters. As main findings, we observed stronger alpha band oscillations in the visual cortex when attention was directed to tactile compared to visual stimuli in both study groups. Furthermore, in patients only, we found stronger beta band oscillations in the right sensorimotor cortex, i.e., contralateral to the tactile stimulation site, when attention was directed to visual as opposed to tactile stimuli. This attention effect on sensorimotor beta oscillations was negatively correlated with positive and cognitive PANSS dimensions in patients.

Our behavioral data support the notion that attention deficits in patients with SZ, which have been observed in unisensory studies^3,46^, extend to IA paradigms. Previously, we reported deficits in IA for the poststimulus processing of multisensory stimuli, as reflected in ERPs^6^. Furthermore, we found that the relatively intact integration of multisensory stimuli in patients can compensate for the deficits in IA, as found at the behavioral level for unisensory but not for bisensory redundant target stimuli. Thus, our behavioral data, together with the previously reported alterations in poststimulus IA processing^6^, suggest IA deficits in patients with SZ.

In terms of neural oscillations, we found in both study groups that alpha and beta band oscillations in the visual cortex were stronger when attention was directed to tactile compared to visual stimuli. Alpha band oscillations in the visual cortex likely have a role in modulating neural excitability and information flow^47,48^. Suppression of visual alpha band activity when attention is directed to the visual modality is a well-established finding^42,49^. Moreover, our previous study in healthy individuals, in which we used an auditory cue to direct attention to the visual or tactile modality, showed relatively stronger visual alpha and beta band activity when attention was directed to the tactile modality^15^, which is in agreement with another study that found stronger alpha oscillations over parieto-occipital scalp when participants attended to auditory compared to visual stimuli^12^. Thus, in the current study, the parallels in attention effects on alpha and beta band activity between groups suggests relatively normal IA effects in patients’ visual cortex.

A different picture of results emerged in the sensorimotor cortex contralateral to the stimulation site. In the control group, we found no significant attention effects on either alpha or beta band oscillations. The lack of attention effects on ongoing sensorimotor oscillations in this group differs from the results of our previous study, in which we observed cue-related attention effects on both alpha and beta oscillations in healthy individuals^15^. However, there was a clear difference in the previous experiment compared to the current experiment. In our previous study, attention was modulated for each trial via an auditory cue, whereas the present study, attention was directed for whole blocks. Thus, for control participants, blockwise up- or down-regulation of ongoing alpha band oscillations in visual cortex may have been sufficient to perform the visual-tactile IA task efficiently, even without additionally modulating alpha or beta band oscillations in the sensorimotor cortex. In the patient group, we found larger sensorimotor beta band oscillations when attention was directed to visual stimuli, compared to when it was directed to tactile stimuli, and this enhancement differed significantly from that in the control group. Our behavioral data as well as findings from previous studies^6,50,51^ show that patients with SZ have deficits in attending to and the ongoing processing of tactile stimuli in the contralateral sensorimotor cortex. In our study, the deficits were found in a task requiring IA. It is possible, that the observed alterations in sensorimotor beta oscillations are related to deficits in ongoing unisensory tactile processing, which have thus far not been explicitly studied in patients with SZ. In contrast to the sensorimotor cortex contralateral to the tactile stimulation site, we did not find IA modulations in alpha and beta band activity in the ipsilateral, i.e., left sensorimotor cortex, suggesting that there were no differences in ongoing motor preparation (as participants responded with the right hand) between attentional blocks.

In our study, we found a negative correlation between the contralateral sensorimotor beta attention effect with cognitive and positive symptoms. Patients with fewer clinical symptoms showed larger beta band attention effects. Gascoyne et al. ^52^ found a reduction in movement related beta fluctuations, which were correlated with a disorganized component of SZ symptomatology. Direct comparison with our own results, in which positive and cognitive dimensions showed the strongest effects, is difficult as different researchers define different dimensions of the PANSS scale^53^. There is some overlap at least with the disorganization defined by Gascoyne et al. ^52^ and our cognitive dimension of SZ.

In our study, the IA effects in the sensorimotor cortex were not predictive of the individual patient performance. This suggests that the attention effects on ongoing sensorimotor beta oscillations, which were negatively correlated with clinical and cognitive symptoms, do not directly influence or compensate for the attention effects at the behavioral level. Previously, we found that IA effects on poststimulus ERPs correlated with the behavioral performance, but not with the cognitive or clinical symptoms in patients^6^. Furthermore, the lack of a relationship between IA effects on ongoing sensorimotor beta oscillations and behavioral performance in patients seems to contradict previous observations in healthy individuals^43,44^ and in animals^54^. However, in these studies, beta oscillations were examined in relation to the task associated with the respective stimulus, in contrast to the current study, in which attention was modulated blockwise. Thus, in patients, alterations in ongoing IA processing, as reflected in sensorimotor beta oscillations, and stimulus processing itself^6^ seem to be related to different aspects of the schizophrenia psychopathology.

Our study has several limitations. Our sample size consisted of a heterogeneous group of participants with schizophrenia, we did not factor in the date of first onset of psychosis. A measure progress of the disorder could have helped understand the variance within the results. Related to heterogeneity of symptoms, the interpretation of neural oscillations and symptom dimensions is dependent on the precision of the PANSS interview. However, there is an ongoing debate on how the symptom dimensions should best be measured and classified^53^. Ideally, we would have a group with little or no mediation. In the current study, our compromise was to select only patients without medications known to substantially affect the EEG signal, and statistically control for the rest. However, we cannot completely rule out the possibility of confounds by medication in the results. It is possible that perceptual differences also affected performance in addition to attention. Future studies could deepen our understanding of this by adding a further control condition with a perceptual task only, or a titration phase to individually adjust to visual/tactile stimulation. In addition, performance on the task was very good across both groups, so we do not know how participants would respond to more challenging attentional modulation tasks. Finally, our understanding of beta oscillations has changed in recent years. The smooth beta oscillations that one sees at the level of averaged trials, can be seen as being composed of discrete beta fluctuations or bursts at the single trial level^55^, including in people with SZ^52^, have been frequently related to integration across cortical regions. Since beta band oscillations appear to be integral to multisensory attention performance in SZ, extending this analysis framework to IA would help us to better understand this phenomenon.

In conclusion, our study shows alterations in ongoing sensorimotor beta oscillations during an intersensory attention task in patients with SZ, which may reflect alterations in neural excitability. The beta-band effect was accompanied by deficits in behavioral performance, particularly the processing of unisensory targets, but not multisensory targets^6^, and was negatively correlated with clinical and cognitive symptoms. Using a visual-tactile stimulation setup, our study suggests that deficits in intersensory attention and alterations in ongoing sensorimotor processing relate to the schizophrenia symptomatology.

## Supplementary information

Supplementary material A: Time course of alpha and beta power for each VOI

Supplement B: Fast Fourier Transform (FFT) and Individualized Alpha control analysis

Supplement C: BACS

## Data Availability

The raw data for this study cannot be shared publicly because of data protection laws and regulations concerning clinical patient data. Limited access to aggregated data and the analytical methods are available upon reasonable request. Researchers interested in accessing these data should contact the corresponding author (JKM). Any requests are subject to approval by the ethics commission and compliance with data protection laws.

## Code availability

All code used for the data analysis is available from the corresponding author (JKM) upon request.
