# Author Correction: Intersensory attention deficits in schizophrenia relate to ongoing sensorimotor beta oscillations

**DOI:** 10.1038/s41537-025-00588-z

**Published:** 2025-02-28

**Authors:** James Kenneth Moran, Daniel Senkowski

**Affiliations:** Department of Psychiatry and Neurosciences, Charité - Universitätsmedizin Berlin, corporate member of Freie Universität Berlin and Humboldt Universität Zu Berlin, Charité Campus Mitte (CCM), Charitéplatz 1, 10117 Berlin, Germany

**Keywords:** Schizophrenia, Biomarkers

Correction to: *Schizophrenia* 10.1038/s41537-025-00571-8, published online 17 February 2025

This article omitted an acknowledgement of support from the German Research Foundation (DFG grant numbers SE1859/4-1 and SE1859/9-1 to D.S.). The original article has been corrected.

