# Supplementary material for: Intersensory attention deficits in schizophrenia relate to ongoing sensorimotor beta oscillations
Source: Schizophrenia (Heidelb). 2025 Feb 17;11(1):19. doi: 10.1038/s41537-025-00571-8 (PMC11832887; doi:10.1038/s41537-025-00571-8)

# Supplementary material A: Time course of alpha and beta power for each VOI

The following graphs show the relative power difference of visual minus tactile attention conditions, normalized with tactile conditions ((V – T)/T)*100, for the right sensorimotor VOI, Left sensorimotor VOI and Occipital VOI. The graphs show the relative power change over the time from -800ms to -200ms before stimulus onset. A parametric running t-test is also carried out for each of the 60 time points measured here, with a critical significance of p < 0.01 marked. The t-values are uncorrected, the idea is to give a supplementary visual impression of the data, to augment our central tests.

Right Sensorimotor VOI:


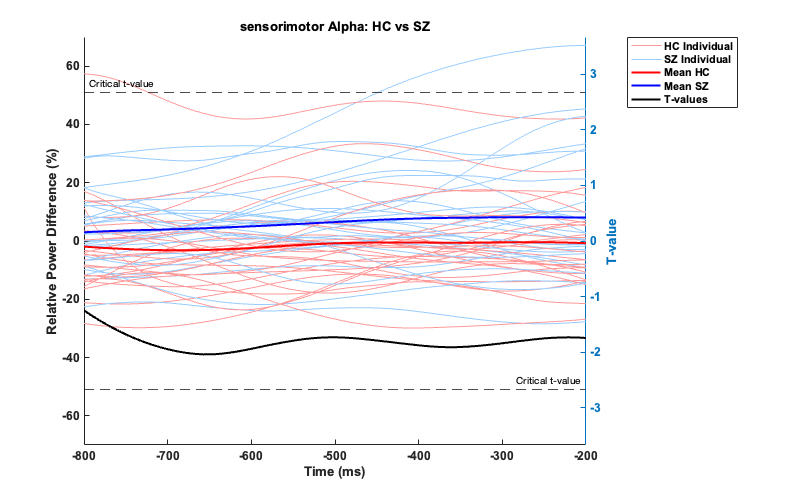


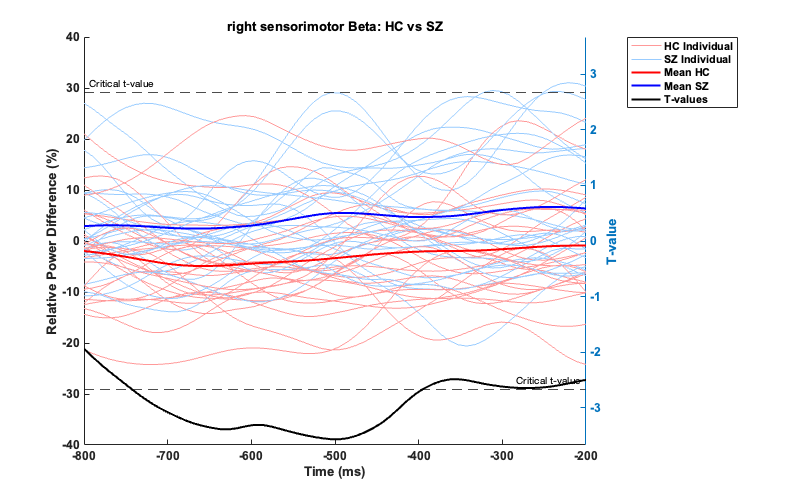


Left sensorimotor VOI:


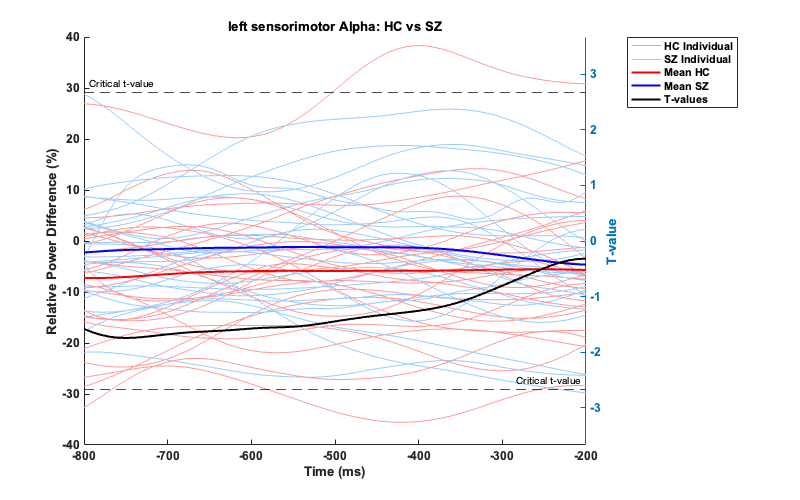


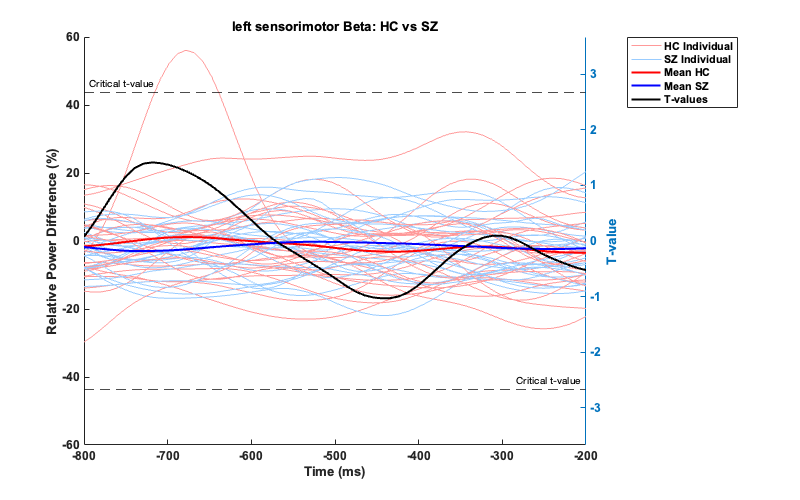


Occipital VOI:


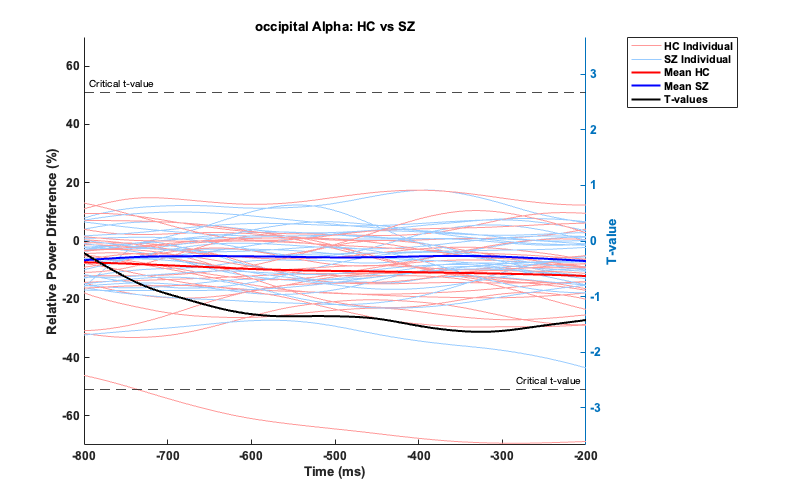


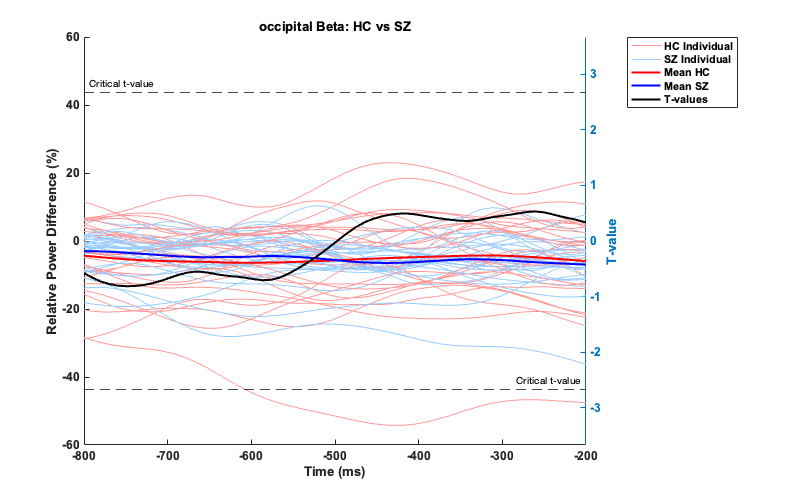

Supplement: Supplementary file 1 — Supplementary material A: Time course of alpha and beta power for each VOI [file 41537_2025_571_MOESM1_ESM.docx]
