# Supplementary material for: Intersensory attention deficits in schizophrenia relate to ongoing sensorimotor beta oscillations
Source: Schizophrenia (Heidelb). 2025 Feb 17;11(1):19. doi: 10.1038/s41537-025-00571-8 (PMC11832887; doi:10.1038/s41537-025-00571-8)
Supplement: Supplementary file 2 — Supplement B: Fast Fourier Transform (FFT) and Individualized Alpha control analysis [file 41537_2025_571_MOESM2_ESM.pdf]

Supplement B: Fast Fourier Transform (FFT) and Individualized Alpha control analysis

To test whether this influenced our individualized alpha for each participant, as some previous studies have suggested differences in the alpha peak frequency between people with SZ and HC [1,2]. This was confirmed in a Fast Fourier Transform (FFT) carried out on the TOI and different FOIs. An LME comparing peak frequency, against Group, Modality and VOI, showed main effect of Group, with SZ (adj-M = 9.91, 9.62-10.20) lower than HC (adj-M = 10.35, 10.06-10.60; $F(1,52) = 4.69$, $p = 0.035$). There was no effect of Attention ($F(1, 260) = 0.11$, $p = 0.744$), or VOI ($F(2, 260) = 2.49$, $p = 0.085$). Thus, the Frequency of Interest (FOI) used for the analysis was defined as the mean individual peak across the nodes of the VOI (occipital: SZ: M = 9.98 SD = 0.942; HC: M = 10.6, SD = 1.06; left sensorimotor: SZ M = 9.70, SD = 1.19; HC: M = 10.2, SD = 1.37; right sensorimotor: SZ M = 10.0, SD = 1.32; HC: M = 10.3, SD = 1.22), averaged across attention conditions for each participant ±2 Hz.

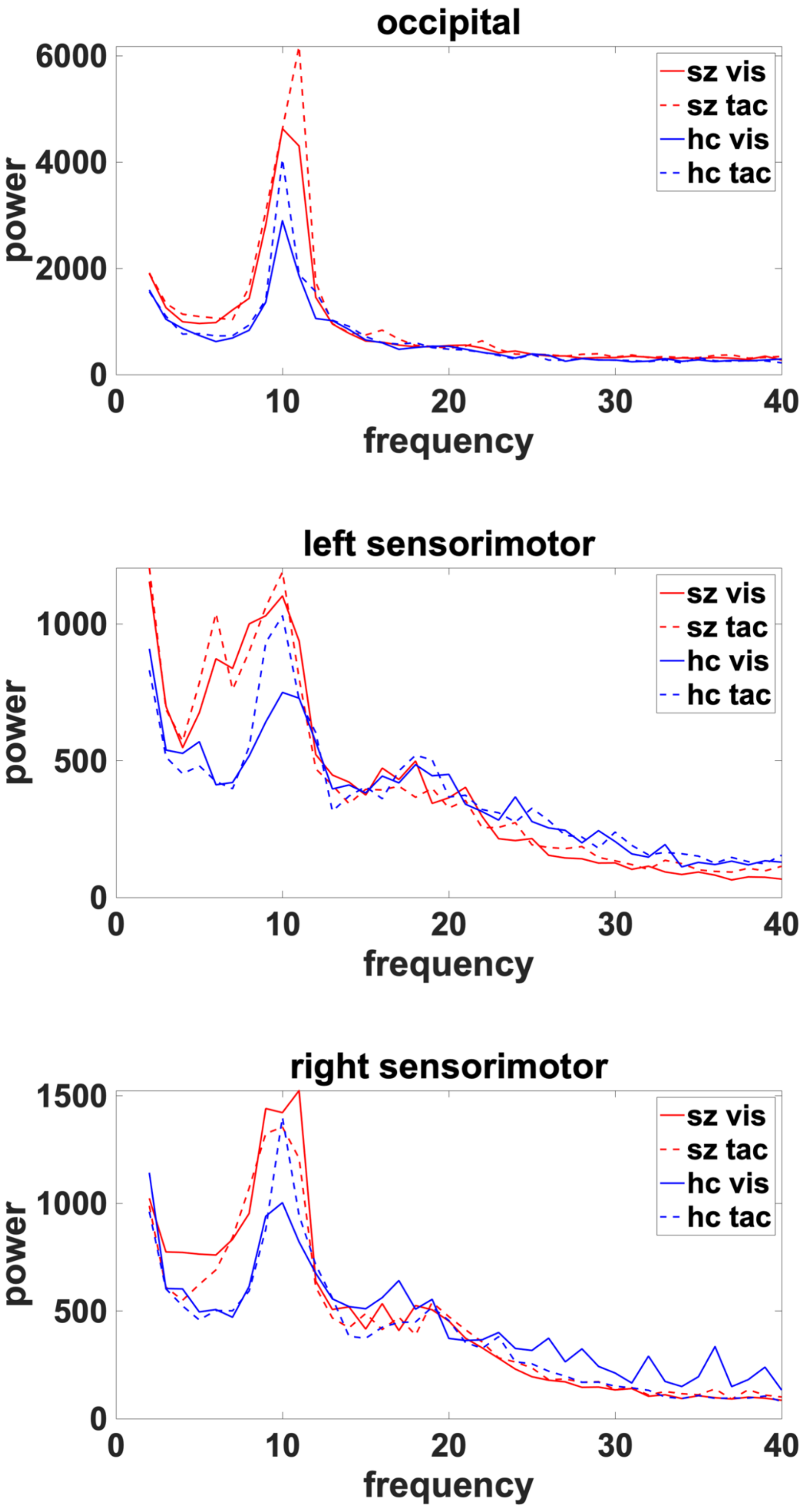

Figure 1 Diagrams of FFTs for each VOI, with Group (SZ red, HC blue), and Attention (Attend-visual continuous, Attend-tactile dotted).

The LME for the HC group showed a significant effect of VOI ($F(2,52) = 11.06$, $p < 0.0001$) with the occipital VOI showing a lower relative alpha power than left sensorimotor VOI ($\beta = -0.009$); and right sensorimotor VOIs ($\beta = -0.36$). Contrasts showed the predicted relative alpha decrease for the HC group in the occipital VOI (adj-M = -9.67, [-14.27, -5.08], $t(42.7) = -4.25$, $p = 0.0003$, BF10 > 100) (Figure 2a).

Examining both groups, we found a main effect of VOI for alpha activity ($F(2, 104) = 16.43$, $p < 0.0001$), but not for group ($F(1, 52) = 3.23$, $p = 0.078$, $\beta = -0.19$). There was no significant interaction between VOI and group ($F(2, 104) = 0.52$, $p = 0.60$). Follow-up Bonferroni adjusted contrasts showed that there was a greater relative change in the right sensorimotor VOI, compared to left (diff-M = -6.46, $t(104) = -3.69$, $p = 0.001$, BF10 = 55.61) and occipital (diff-M = -9.88, $t(104) = -5.65$, $p < 0.0001$, BF10 > 100) VOIs, with no evidence of difference between left and occipital VOIs (diff-M = 3.42, $t(104) = 1.96$, $p = 0.16$, BF10 = 0.68) (Figure 2b).

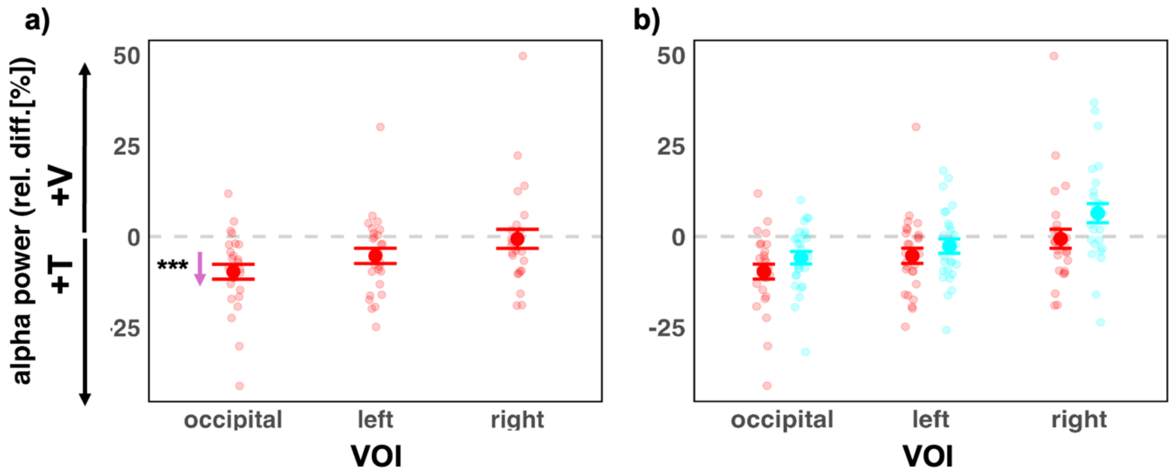

*Figure 2. a) Replication of the alpha power differences in the HC group with individualized alpha peaks, defined as the individual peak ±2 Hz. The difference from zero was calculated for each VOI with Bonferroni adjusted contrasts, showing the relatively stronger alpha activity in the attend-tactile condition in the occipital region. b) individualized alpha for both groups. Here there were no group differences or interactions. ***p < 0.0001.*

References:

1. Murphy, M., & Öngür, D. (2019). Decreased peak alpha frequency and impaired visual evoked potentials in first episode psychosis. NeuroImage: Clinical, 22, 101693.

2. Ramsay, I. S., Lynn, P. A., Schermitzler, B., & Sponheim, S. R. (2021). Individual alpha peak frequency is slower in schizophrenia and related to deficits in visual perception and cognition. Scientific reports, 11(1), 17852.
