# Supplementary material for: Intersensory attention deficits in schizophrenia relate to ongoing sensorimotor beta oscillations
Source: Schizophrenia (Heidelb). 2025 Feb 17;11(1):19. doi: 10.1038/s41537-025-00571-8 (PMC11832887; doi:10.1038/s41537-025-00571-8)
Supplement: Supplementary file 3 — Supplement C: BACS [file 41537_2025_571_MOESM3_ESM.pdf]

Supplement C: BACS

A regression of BACS total scores (z-normed) against group and relative Beta Power chanbge, showed that relative beta power changes predicted a reduction in BACS scores, and the SZ group had a much lower BACS than the HC group (See Table 1). The interaction was significant in this, however, inspection of the graph appeared to show that the beta power*group interaction was driven by one extreme value in the HC group (See Fig. 1). Redoing the regression without this value removed the beta power*group interaction, the other main effects remained however.

Table 1 regression of beta power and group on BACS composite z-score. The upper table represents the full dataset, the lower table represents the dataset with the influential HC datapoint removed, showing that the interaction is no longer significant.

BACS (composite z-score)			
**Original**	**beta**	**F(1,50)**	**p**
beta power (rel. diff [%])	-0.17	6.66	0.013*
group	0.37	8.17	0.006**
beta power*group	-0.27	4.27	0.044*
**Outlier removed**	**beta**	**F(1,49)**	**p**
beta power (rel. diff [%])	-0.15	5.07	0.029*
group	0.36	7.57	0.008**
beta power*group	-0.26	3.55	0.066

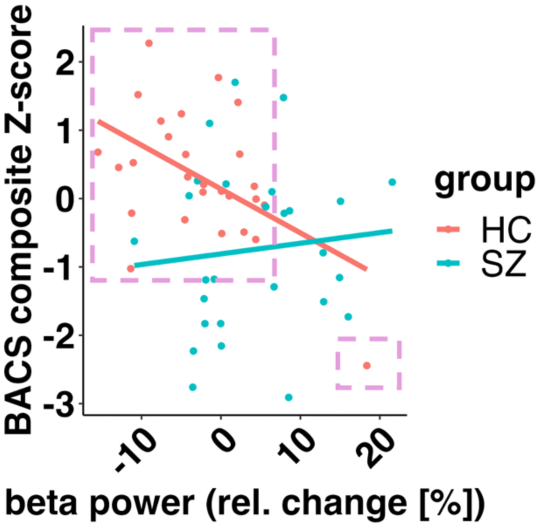

Figure 1 Scatterplot of beta power and BACS scores, separately for group (HC and SZ). The purple boxes show the clustering of the HC in the upper left and the single HC influential point in the bottom right, respectively.
